# Proceedings of the Twelfth Annual Meeting, Fourteenth Anniversary, of the Illinois State Medical Society

**Published:** 1864-06

**Authors:** 


### Proceedings of Societies.

### PROCEEDINGS OF THE TWELFTH ANNUAL MEETING, FOURTEENTH ANNIVERSARY, OF THE ILLINOIS STATE MEDICAL SOCIETY.

HELD IN CHICAGO, MAY THIRD, FOURTH, & FIFTH, 1864.

The Fourteenth Anniversary Meeting of the Illinois State Medical Society, assembled in the Common Council Room, in the City of Chicago, on the morning of May 3d, 1864. In the absence of the President, Dr. A. H. Luce, of Bloomington, first Vice-President, took the Chair and called the Society to order at 10½ o'clock A.M.

Dr. M. O. Heydock, of Chicago, Chairman of the Committee of Arrangements, welcomed the members of the Society to the hospitalities of the city, in a brief and very appropriate address, and reported the following list of permanent members and delegates as present, viz.:—

Dr. H. Noble, of Heyworth, McLean Co., Ill.
" A. H. Luce, of Bloomington, " "
" J. S. Whitmire, of Metamora, Woodford Co., Ill.
" David Prince, of Jacksonville, Morgan Co., "
" L. T. Hewins, of Loda, Iroquois Co., "
" M. O. Heydock, of Chicago, Cook Co., "
" E. L. Holmes, " " "
" W. H. Byford, " " "
" N. S. Davis, " " "
" Gerhard Paoli, " " "
" R. E. McVey, of Waverly, Sangamon Co., "
" S. Wickersham, of Chicago, Cook Co., "
" E. Andrews, " " "
" J. H. Hollister, " " "
" Ira Hatch, " " "
" DeLaskie Miller, " " "
" John Bartlett, " " "

" Thomas Bevan, " " "
" R. N. Isham, " " "
" J. M. Steele, of Grandview, Edgar Co., "
" E. Ingalls, of Chicago, Cook Co., "
" D. W. Young, of Aurora, Kane Co., "
" Chas. G. Smith, of Chicago, Cook Co., "
" Alex. Fisher, " " "
" J. Adams Allen, " " "
" D. E. Ellis, of Belvidere, Boone Co., "
" Noble Holton, of Buda, Bureau Co., "
" John Woodworth, of Aurora, Kane Co., "
" N. P. Peterson, of Chicago, Cook Co., "
" J. W. Freer, " " "
" V. L. Hurlbut, " " "
" J. P. Ross, " " "
" J. S. Jewell, " " "

The Secretary then called the full roll of members; and, on motion, the reading of the minutes of the last Annual Meeting was dispensed with.

The election of permanent members being in order, the following were duly proposed and elected, as new members:—

Drs. R. C. Hamill, of Chicago; A. Groesbeck, of Chicago; Henry M. Lyman, of Chicago; James Miner, of Waverly; Noble Holton, of Buda; L. F. Warner, of Chicago; E. P. Cook, of Mendota; J. P. Ross, of Chicago; H. Wing, of Chicago; J. H. Foster, of Libertyville; J. H. Hagerty, of Dwight; F. N. Burdick, of Elgin; Joseph Tefft, of Elgin; S. C. Blake, of Chicago; J. S. Jewell, of Chicago; James H. O'Reilly, Chicago; W. R. Fox, of Wilmington; M. J. Johnson, of Wilmington; G. B. Lester, Bristol; F. A. Emmons, of Aurora.

On motion of Dr. J. H. Hollister, a committee of one from each county represented, was appointed to nominate officers and Standing Committees for the ensuing year.

The following were announced as such Committee:—

H. Noble, of McLean Co.
R. E. McVey, of Morgan Co.
R. C. Hamill, of Cook "

J. M. Steele, of Edgar "
J. S. Whitmire, of Woodford Co.
L. T. Hewins, of Iroquois "
D. W. Young, of Kane "
E. P. Cook, of Lasalle "

On motion of Dr. S. Wickersham, Surgeon S. H. Holden, U.S.A. and Medical Director for Chicago, and Surgeon J. H. Grove, U.S.A., Superintendent of General Hospitals of Chicago, were elected members by invitation.

On motion of Dr. E. L. Holmes, W. H. Baxter, of Moscow, Iowa, was elected a member by invitation.

Dr. M. O. Heydock, Chairman of the Committee of Arrangements, presented invitations from the faculty of the Chicago Medical College; the Rush Medical College; the Marine and Mercy Hospitals; and from the Surgeons of the Military Hospitals in Camp Douglas, to visit each of those institutions and recommended that the several invitations be accepted, and that a part of the afternoon of Wednesday be devoted to the visiting of Rush Medical College and the Marine Hospital; and the whole of the afternoon of Thursday to the visiting of the Chicago Medical College, and the Mercy and Military Hospitals.

On motion, the recommendations of the Committee were unanimously adopted.

An invitation was received from Dr. N. S. Davis, requesting the members of the State Medical Society to meet those of the Chicago City Medical Society in a social entertainment at his residence on Wednesday evening, which was accepted. The Treasurer, Dr. J. H. Hollister, of Chicago, submitted the following report, which was accepted and approved:—

J. H. Hollister, *Treasurer*,

In ac't with Ill. State Med. Society,

| | |
|---|---|
| 1683. *Dr.* | |
| May 7.—To Cash received from new members at Jacksonville, May 7th, 1863, for Initiation, | $22 00 |
| Cash of the same—dues for 1863 - - - | 20 00 |
| Cash received from old members—dues 1863, | 28 00 |
| | $70 00 |

1863. *Cr.*

May 12.—By amount paid Wm. Craven, Printer—
Balance of Bill for Transactions, 1860, - - $69 50

$00 50

Amount due G. H. Fergus, Printer, for publishing the Transactions for 1863, for which the Society is now indebted, - - - - - - 56 05

Respectfully submitted.

J. H. HOLLISTER, *Treas.*

Dr. D. W. Young, of Aurora, moved that the annual assessment for the present year be three dollars, which motion, after some discussion, was adopted.

The Permanent Secretary, Dr. N. S. Davis, submitted the following annual report:—

REPORT OF THE COMMITTEE OF PUBLICATION AND SECRETARY FOR 1864.

Within sixty days after the adjournment of the last Annual Meeting, the undersigned notified, by letter, all the Committees appointed at that meeting.

As the cash receipts of the last year were no more than sufficient to pay the previous indebtedness of the Treasury, leaving nothing to pay for the Transactions of the past year, the Secretary had no alternative but to forego the publication of the Transactions altogether, or pursue the same course as in previous years, namely, first publish the reports and papers in the Medical Examiner, and thereby avoid all the expense of type-setting—leaving the tax upon the Treasury of the Society to consist only of paper and press-work.

The Publishing Committee chose the latter course, and published 300 copies of the Transactions of the Meeting at Jacksonville, at a cost of $68. Fifty copies were taken by Dr. Prince, of Jacksonville, and paid for, *pro rata*, as the cost of publication, leaving a balance of $56.05. Copies of the Transactions have been mailed to all the members who had paid the annual assessment for the year—leaving about 200 copies now on hand—about 30 of which have been laid on the table in this room for the use of members.

In behalf of the Committee of Publication.

N. S. DAVIS,
*Per. Secretary.*

On motion of Dr. DeLaskie Miller, the report was accepted and the thanks of the Society tendered to the Secretary for his liberality in procuring the publication of the transactions.

Reports of Standing Committees being called for, the Chairmen of several announced their possession of reports; and, after fixing the time for hearing the same, the Society adjourned to 2 o'clock P.M.

AFTERNOON SESSION.

At 2 o'clock P.M., the Society was called to order, Dr. A. H. Luce, Vice-President, in the chair.

Dr. H. Noble, Chairman of Committee on Nominations, reported as follows:—

*For President.*—A. H. LUCE, M.D., of Bloomington.

*Vice-Presidents.*—J. M. STEELE, M.D., of Grandview; THOMAS BEVAN, M.D., of Chicago.

*Assistant-Secretary.*—C. R. PARKE, M.D., of Bloomington.

*Treasurer.*—J. H. HOLLISTER, M.D., of Chicago.

*Committee of Arrangements.*—T. F. WORRELL, M.D., of Bloomington; S. W. NOBLE, M.D., of LeRoy; T. P. ROGERS, M.D., of Bloomington.

*Committee on Practical Medicine.*—J. ADAMS ALLEN, M.D., of Chicago; C. GOODBRAKE, M.D., of Clinton; H. NOBLE, M.D., of Heyworth.

*Committee on Drugs and Medicines.*—JOHN BARTLETT, M.D., of Chicago; F. R. PAYNE, M.D., of Marshall; T. D. FITCH, M.D., of Kewanee.

*Committee on Obstetrics.*—WM. H. BYFORD, M.D., of Chicago; LUCIUS CLARK, M.D., of Rockford; S. T. TROWBRIDGE, M.D., of Decatur.

*Committee on Surgery.*—D. W. Young, M.D., of Aurora; A. L. MCARTHUR, M.D., of Joliet; L. T. HEWINS, M.D., of Oakalla.

*Special Committee on Ophthalmology.*—E. L. HOLMES, M.D., of Chicago; R. E. MCVEY, M.D., of Waverly; J. S. WHIT-

MIRE, M.D., of Metamora; F. B. HALLER, M.D., of Vandalia.

*Special Committee on Spotted Fever.*—J. S. JEWELL, M.D., of Chicago.

*Special Committee on Orthopœdic-Surgery.*—DAVID PRINCE, M.D., of Jacksonville.

Bloomington was recommended as the place for holding the next Annual Meeting of the Society.

On motion, the report of the committee was accepted; and the officers and committees nominated therein, unanimously elected. The President elect returned thanks to the Society for the honor conferred.

Letters from absent members being in order, the Secretary read a letter from the retiring President, Dr. A. McFarland, Superintendent of Hospital for Insane at Jacksonville, expressing his warm attachment to the Society, and his regret at not being able to attend the meeting on account of the sickness of his only Assistant in the Hospital.

On motion of Dr. J. A. Allen, Dr. McFarland was requested to furnish a copy of his Annual Address to the Publishing Committee for publication in the Transactions of the Society.

The Secretary also read a letter from Prof. H. A. Johnson, of Chicago, regretting his inability to attend the meetings of the Society on account of severe illness.

The reading of volunteer papers being in order, Dr. R. E. McVey, of Waverly, read an interesting communication on cerebro-spinal meningitis or spotted fever, as it had prevailed in the circle of his own practice.

Dr. J. Adams Allen, of Chicago, also read a very interesting paper on the same subject, referring to a severe epidemic of the disease, which came under his observation in 1848–9.

These communications were listened to with much interest; and, after a brief discussion by Drs. Hollister, Andrews, Whitmire, and Allen, the authors were requested to furnish copies of their respective papers to the Committee of Publication.

Dr. E. Andrews, of Chicago, Chairman of the Standing Committee on Surgery for the past year, read an abstract of his report, which was accepted and a full copy of the report referred to the Committee of Publication.

Dr. E. L. Holmes, of Chicago, read an abstract of his report as Special Committee on diseases of the eye. On motion of Dr. J. S. Whitmire, the report was accepted and referred to the Committee of Publication.

Dr. E. Andrews, of Chicago, Chairman of the Auditing Committee appointed at the last Annual Meeting, made the following report, which was adopted, viz.:—

"The Committee to which was referred the Annual Report of the Treasurer, Dr. J. W. Freer, beg leave to report that they have investigated the same, and found it to be correct, and recommend its approval.

E. ANDREWS,
*Chairman of Com.*"

Dr. N. S. Davis offered the following resolution, which was adopted, viz:—

*Resolved*, That all reports of committees and volunteer communications, referred to the Committee of Publication, must be complete and delivered to the Permanent Secretary before the first of July next succeeding the meeting at which they were read.

On motion, the Society adjourned to 9½ o'clock in the morning.

MAY FOURTH—SECOND DAY.

Wednesday morning, 9½ o'clock, the Society was called to order,—Dr. A. H. Luce, President, in the chair.

The Secretary read the minutes of yesterday, which were approved. The Society also read a communication from Dr. F. K. Bailey, formerly of Joliet, but now in the U. S. General Hospital at Quincy, regretting his inability either to attend the meeting or send a report from the Committee on Drugs and Medicines.

Dr. J. M. Steele moved that the Secretary be appointed a Committee to present suitable resolutions in relation to the death of the late Dr. S. York, of Paris, Edgar Co. The motion was seconded and adopted.

The hearing of reports of Standing Committees being resumed, Dr. DeLaskie Miller, of Chicago, read the report of the

Standing Committee on Obstetrics and Diseases of Females. The report elicited an interesting discussion, which was participated in by Drs. Heydock, Prince, Steele, Bartlett, Whitmire, Andrews, and Hamill.

On motion of Dr. Prince, the report was accepted and referred to the Committee of Publication.

Dr. Bartlett offered the following resolution, which was seconded and adopted, viz.:—

*Resolved*, That the Chairman of the late Committee on Obstetrics (Dr. DeLaskie Miller, of Chicago) be appointed a Special Committee to consider the question of the influence of the mind of the mother upon her offspring; and also to inquire whether the husband of a pregnant woman ever experiences morning sickness in her stead.

On motion, Dr. W. H. Byford, of Chicago, was appointed a Standing Committee on Necrology.

Dr. D. Prince, of Jacksonville, presented a pretty full verbal abstract of his report on Orthopœdic Surgery, accompanied by many interesting illustrations.

Before completing his remarks, the hour arrived for adjournment. Dr. Heydock, from the Committee of Arrangements, reported the names of additional members. Adjourned to 2 o'clock P.M.

AFTERNOON SESSION.

Society was called to order at 2 o'clock P.M.,—the President in the chair.

Dr. Prince resumed and completed his report, which was accepted and referred to the Committee of Publication.

At 3½ o'clock P.M., the Society adjourned until 9½ o'clock the following morning, to enable the members to visit Rush Medical College and the Marine Hospital.

THURSDAY, MAY FIFTH—THIRD DAY.

The Society was called to order at 9½ o'clock, A.M.,—the President in the chair.

Secretary read the minutes of the proceedings of yesterday, which were approved.

Dr. J. Bartlett read a letter from Dr. McGugin, of Iowa, on the subject of the fees of medical witnesses, and offered the following resolutions, which were adopted:—

*Resolved,* That a committee of three be appointed by the Chair, to consider in what respects the pecuniary interests of the medical profession suffer from unfavorable or deficient legislation.

*Resolved,* also, That this committee be authorized to petition the State Legislature, in behalf of this Society, for such legislation as they may deem necessary and proper.

Dr. N. S. Davis reported the following resolutions in relation to the death of Dr. S. York, which were unanimously adopted:

*Whereas,* Dr. S. York, of Paris, Edgar Co., Ill., an honored and highly esteemed member of this Society, has been recently stricken down, while in the service of his country, by the hand of lawless violence; therefore,

*Resolved,* That by his death, this Society has lost one of its most faithful, active, and honored members; the community where he lived, one of its most skilful physicians; and the country, one of its most patriotic citizens.

*Resolved,* That we deeply sympathize with his afflicted family and friends in this hour of their bereavement.

*Resolved,* That the Secretary be directed to forward a copy of the foregoing resolutions to the family of the deceased.

The President appointed the following committee to act on the subject of Dr. Bartlett's resolutions, and report at the next Annual Meeting, viz.:—Drs. John Bartlett, of Chicago; A. McFarland, of Jacksonville; and H. Wing, of Chicago.

Dr. D. W. Young offered the following resolutions, which were adopted, viz.:—

*Resolved,* That the present pay and rank of surgeons and assistant-surgeons in the army is inadequate to compensate them for the services required of and performed by them.

*Resolved,* That the members of this Association ought to make every possible exertion, through the National Medical Association, and our senators and representatives in Congress, to have our medical brethren in the field receive at least a reasonable compensation for their services and sacrifices while they are braving the dangers of the camp and caring for the soldiers of our country.

Dr. H. Wing offered the following resolutions, which were referred to the committee appointed under the resolutions of Dr. Bartlett:—

*Resolved*, That the Profession is the proper judge of the qualifications of its members.

That the interests of the Profession would be promoted by having new members admitted on the judgment of the Profession itself; and, therefore,

That the committee appointed under resolution, to memorialize the State Legislature, be also instructed to ask that this Society be authorized by law to grant certificates of qualification, on proper examination, to undergraduates.

Dr. N. S. Davis, Chairman of Committee on Practical Medicine, read a somewhat lengthy report, which was accepted and referred to the Committee of Publication.

Dr. J. Woodworth offered the following, which was unanimously adopted:—

Mr. President:—As the hour for adjournment of this Society has arrived, and this is the last opportunity which will present itself wherein we can as a body recognize the courtesy extended to us by individual members of the profession during its session; I would therefore move that the thanks of this Society be expressed to the Common Council, the Faculty of the Rush Medical College, the Faculty of the Medical Department of the Lind University, the Physician in charge of the Mercy Hospital, and especially to Drs. R. N. Isham and A. R. Terry, of the U. S. Marine Hospital; Dr. J. H. Grove, U.S.A. Superintendent of Hospitals; Dr. Holden, U.S.A. Medical Director; and Dr. N. S. Davis, of Chicago, for those courtesies which have done so much to render this meeting one of interest, pleasure, and profit.

On motion, the following members were elected delegates to the American Medical Association for the ensuing year:—

Dr. N. Wright, of Chatham.
" D. Prince, of Jacksonville.

" R. E. McVey, of Waverly.
" H. Noble, of Heyworth.
" N. S. Davis, of Chicago.
" D. W. Young, of Aurora.
" J. M. Steele, of Grandview.
" J. Woodworth, of Aurora.
" J. Bartlett, of Chicago.
" J. S. Jewell, of Chicago.
" L. Clark, of Rockford.
" R. C. Hamill, of Chicago.
" N. Holton, of Buda.
" A. L. McArthur, of Joliet.
" E. P. Cook, of Mendota.
" T. D. Fitch, of Kewanee.
" S. W. Noble, of LeRoy.
" C. Goodbrake, of Clinton.

At 12½ o'clock, the Society adjourned for the year—it being understood that the afternoon should be occupied in visiting the Chicago Medical College, Mercy Hospital, and the Military Hospitals in Camp Douglas.

N. S. DAVIS, *Per. Secretary.*

H. W. JONES, *Assist.* "

